# Born to Run Out of COVID-19: What Gives Us Wings

**DOI:** 10.3389/fspor.2020.00105

**Published:** 2020-08-25

**Authors:** Luca Filipas, Antonio La Torre, Livio Luzi, Roberto Codella

**Affiliations:** ^1^Department of Biomedical Sciences for Health, Università degli Studi di Milano, Milan, Italy; ^2^Department of Endocrinology, Nutrition and Metabolic Diseases, IRCCS MultiMedica, Milan, Italy; ^3^Italian Athletics Federation, Rome, Italy

**Keywords:** global health, physical activity, Olympics, endurance running, metabolism

## When Covid-19 Exacerbates Another Pandemic: Physical Inactivity

A pandemic called COVID-19 has spread worldwide in the first months of 2020. Several measures have been taken by mostly affected countries to contain the virus outbreak, from social distancing to closing social and commercial activities. Also, sports dynamics has been dramatically impacted by this pandemic, with training and competitions canceled and no clear hypotheses on the expected restart dates. The International Olympic Committee even postponed Tokyo 2020 Olympics to 2021, with lots of consequences on athlete's preparation and mental focusing. Long-distance runners have seen canceled or postponed all the spring races, included the Major Marathons, and associated economic sequalae are real.

This pandemic could potentially have a huge impact not only on elite athletes' preparation for the Olympics, but also for global health. In fact, the impossibility to engage in regular activities (e.g., school, work, fitness facilities) and utilize community resources (e.g., parks, playgrounds, walking trails) have caused a sharp reduction in the levels of physical activity, and has upended western societies lifestyles. In turn, this may result in a substantially increased risk of developing cardiovascular and metabolic diseases. Indeed, physical activity is associated with reduced risk of mortality and incident cardiovascular and metabolic diseases in all regions of the world, with no indication of a ceiling effect for higher doses. Therefore, the scientific community rated physical activity as a low-cost approach to reducing deaths and cardiovascular disease that is applicable globally with potential large impact (Lear et al., 2017).

## Poor Mental Health and Physical Inactivity: Two Hits From Covid-19

Other factors, such as fear of social contacts and the inability to carry out group activities, could contribute to physical inactivity even once community resources will be reopened. Hence, the prevalence of physical inactivity may rise tragically in the upcoming months, as the social distances measures are expected to be extended at least until summer or autumn. To the best of our knowledge, no studies assessed the long-lasting effect of such a pandemic on physical activity behaviors. However, previous research has assessed the persisting effect of natural disasters on it. As an example, following the 2011 earthquake and tsunami in East Japan, Okazaki and colleagues (Okazaki et al., 2015) reported a significant decrease in physical activity up to 3 years following the disaster.

The evidence base for the relationship between physical activity and mental well-being is well-established (Mason and Kearns, 2013). A large body of literature has consistently shown that physical activity is positively associated with increased mental well-being (Bize et al., 2007; Cerin et al., 2009). In a recent review on psychological impact of quarantine periods, Brooks and colleagues compared post-traumatic states such as stress, depression, and confusion, with experiencing epidemic outbreaks (Brooks et al., 2020). Therefore, a COVID-19-associated reduction in physical activity could impair physiology as well as generate worrying effects at psychological and psychosocial level (Harris, 2018).

## An Alternative Response To The Outbreak

Sustained physical inactivity and sedentary behaviors are typically accompanied by poor physical and mental health and increased disease-specific and all-cause mortality risk (Booth et al., 2017). Even brief periods of exposure to these behaviors can be deleterious; for example, a 2-week reduction in daily steps from ~10,000 to ~1,500 steps led to impaired insulin sensitivity and lipid metabolism, increased visceral fat and decreased fat-free mass and cardiovascular fitness in healthy adults (Krogh-Madsen et al., 2014).

Despite acknowledging the tremendous impact of COVID-19 emergency, the authors would like to highlight potential long-term effects of a sedentary lifestyle that could even cause worse consequences than the infection itself. In this regard, the spread out of the COVID-19 has been greater in the high-income countries, that have at the same time the highest prevalence of physical inactivity (WHO, 2016). A further increase in physical inactivity could have potential drastic effects not only in the onset of cardiovascular and metabolic diseases, but also, in the long-term trends, in sport- performances and results.

## Sociocultural and Lifestyle Habits Could Preserve Metabolism and Lead To Olympic Medals

**Figure 1:**
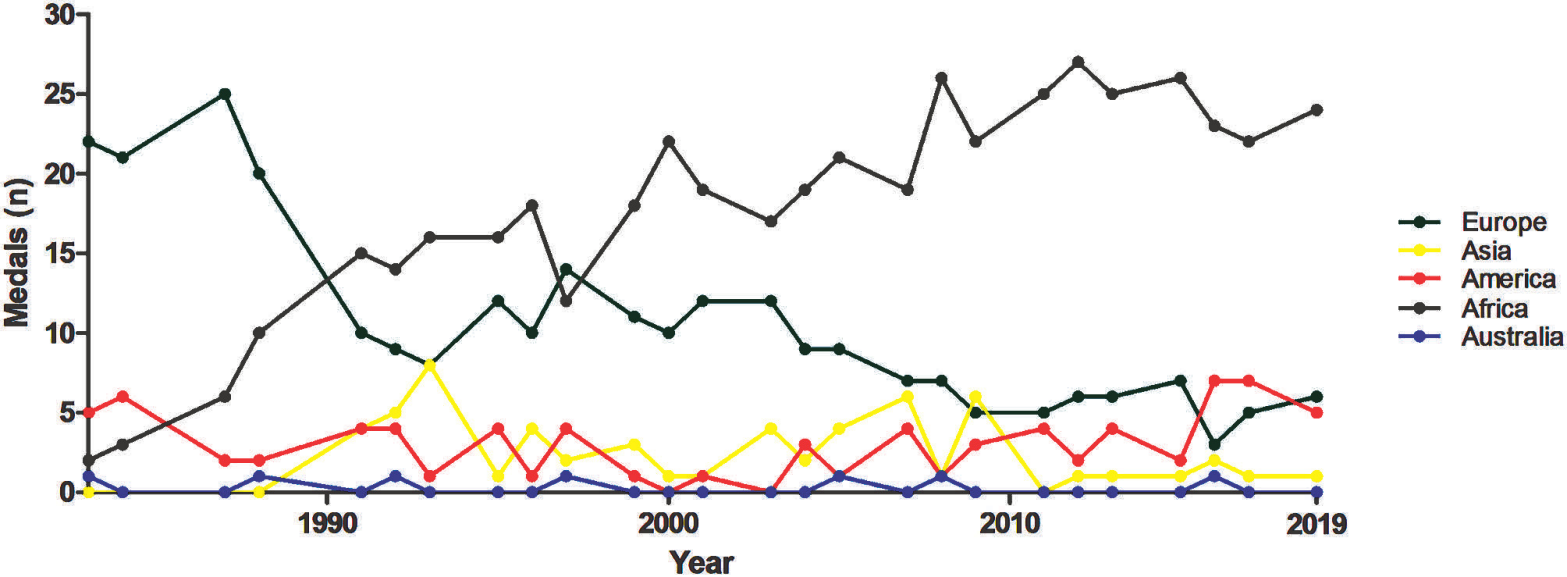
Medals in middle- and long-distance events of athletics at the Olympics and World Championships from 1983 to 2019. Country are grouped in macro-areas (i.e., Africa, America, Asia, Australia, and Europa).

Long-distance athletics could be a mirror of this situation and the dominance of east-African runners at the Olympics and World Championships during the last years provides hints in a global health prospective. In the top-50 all-time performances of the track distance events (from 800 m upward), east Africans men and women passed from 11.4% in 1989 to 61% in 2019, mostly Kenyans, Ethiopians and, even recently, Ugandans. The dominance in the top 50 middle- and long-distance races reflected a spurt in the number of medals at the Olympics and World Championships (Tucker et al., 2015). On the other side, the increase in middle- and long-distance athletics medals at the Olympics and World Championships by Africans athletes was paralleled by the decrease of Europeans' ones, from 1983 (i.e., the first edition of the World Championships) onwards (Figure 1).

The association between physical performance in long-distance athletics competitions and global health prospective finds supports in the 2016 report of the World Health Organization (WHO, 2016). This report showed that the prevalence of insufficient physical activity rises worldwide according to the level of income: high income countries had more than doubled this prevalence compared to low income countries for both men and women (i.e., 32 and 42% of insufficient physical activity in high income compared to 13 and 19% in low income countries, respectively, for men and women). Specifically, if we look at the physical inactivity levels in the east Africa and in particular in Kenya, official reports show a strong difference between children from urban and rural areas, where successful Kenyan runners were born and raised (Onywera et al., 2016). The average levels of children's physical activity in some regions of Kenya are well-above the recommended threshold of 60 min of daily moderate-vigorous physical activity, with more than 150 min spent in free-living physical activity (Ojiambo et al., 2013). Conversely, in western countries more than 80% of adolescents do not reach the claimed minimum level. Moreover, while 13.1% of children of urban areas spend more than 11 h per week playing screen games, instead 62.5% of the children of rural areas spend no time in these activities (Onywera et al., 2012). Increased urbanization in high income countries has resulted in several negative environmental factors such as violence, high-density traffic, low air quality, pollution, lack of parks, sidewalks, and sports/recreation facilities (Manferdelli et al., 2019). This may discourage participation in physical activity along with making adults resistant to leave children play freely outside. Interestingly, while the levels of physical activity are decreasing among children and adults in high income countries and in urban areas, the bound between cardiorespiratory fitness and high levels of physical activity is seemingly reinforced in those emergent countries where fortunate endurance performances are registered (Santos-Lozano et al., 2017).

As this gap is growing further when comparing low- and high-income countries, the next Olympics might confirm east Africans as the favorites for medals in long-distance athletics races. Moreover, the Olympic races show only a part of the phenomenon as, in these events, the number of athletes for each nation is limited to three, thus allowing athletes from other nations to place themselves in prominent positions although their overall rank is lower. As an example, in the 2019 men's marathon lists, the first athlete not born in east Africa is ranked 34th whereas the first athlete born outside of Africa is ranked 45th.

Only by changing some modern detrimental habits and putting again physical activity as pivotal in our lifestyles, we might be able to revert this negative trend, for which high income countries increase exponentially metabolic and cardiovascular diseases while they reduce competitiveness in endurance events.

## Concluding Remarks

Results and performances are not only “sports outcomes,” but also offer an ideal context for understanding deep socio-cultural processes rooted in the development of a country. The lack of a date for the next Olympics is certainly a sports problem, but could have a translation in what is around the world of sport: the example of athletics long-distance events are a clear indicator of how a healthy lifestyle can allow a country to remain at the top of the sport for decades, generating benefits for the health system. As early-life high levels of maximum aerobic capacity are linked to protection from coronary heart diseases (Batty and Lee, 2004), they likewise introduce a sociocultural factor that is determinant, amongst the others, for the success in running competitions. Beyond the extraordinary athletic aptitudes, the strong psychological motivation to succeed is the discriminating factor for any-country runners (Wilber and Pitsiladis, 2012). On the other hand, watching athletes from your country winning medals in international competitions can be a strong incentive to physical activity. In this sense, the Olympics are the fundamental engine of a champion emulation process which lays the foundations for a growth of a national sports movement and, consequently, an increase in the level of physical activity of the nation (Thomas et al., 2019). Elite sport has the potential role to be the psychosocial driving force in pushing the population through a regular practice of physical exercise. We urge public health authorities and community at large not to leave sport at the short end of the stick in this emergency, considering the long-term deleterious effects that a lack of physical activity could cause at metabolic, cardiovascular, psychological, and social levels.

## Author Contributions

LF, AL, LL, and RC participated in conception and design and critically revised the manuscript. LF completed the acquisition of data. LF and RC carried out the analysis, interpretation of data, and participated in drafting of the manuscript. All authors have read and approved the final version of the manuscript and agree with the order of presentation of the authors.

## Conflict of Interest

The authors declare that the research was conducted in the absence of any commercial or financial relationships that could be construed as a potential conflict of interest.
